# Susceptibility of Nrf2-Null Mice to Steatohepatitis and Cirrhosis upon Consumption of a High-Fat Diet Is Associated with Oxidative Stress, Perturbation of the Unfolded Protein Response, and Disturbance in the Expression of Metabolic Enzymes but Not with Insulin Resistance

**DOI:** 10.1128/MCB.00677-14

**Published:** 2014-09

**Authors:** Paul J. Meakin, Sudhir Chowdhry, Ritu S. Sharma, Fiona B. Ashford, Shaun V. Walsh, Rory J. McCrimmon, Albena T. Dinkova-Kostova, John F. Dillon, John D. Hayes, Michael L. J. Ashford

**Affiliations:** a Medical Research Institute, Division of Cardiovascular and Diabetes Medicine, Ninewells Hospital and Medical School, University of Dundee, Dundee, Scotland, United Kingdom; b Division of Cancer Research, Ninewells Hospital and Medical School, University of Dundee, Dundee, Scotland, United Kingdom; c Department of Pathology, Ninewells Hospital and Medical School, Tayside NHS Trust, Dundee, Scotland, United Kingdom

## Abstract

Mice lacking the transcription factor NF-E2 p45-related factor 2 (Nrf2) develop more severe nonalcoholic steatohepatitis (NASH), with cirrhosis, than wild-type (*Nrf2*^*+/+*^) mice when fed a high-fat (HF) diet for 24 weeks. Although NASH is usually associated with insulin resistance, HF-fed *Nrf2*^−/−^ mice exhibited better insulin sensitivity than HF-fed *Nrf2*^*+/+*^ mice. In livers of HF-fed mice, loss of Nrf2 resulted in greater induction of lipogenic genes, lower expression of β-oxidation genes, greater reduction in AMP-activated protein kinase (AMPK) levels, and diminished acetyl coenzyme A (CoA) carboxylase phosphorylation than in the wild-type livers, which is consistent with greater fatty acid (FA) synthesis in *Nrf2*^−/−^ livers. Moreover, primary *Nrf2*^−/−^ hepatocytes displayed lower glucose and FA oxidation than *Nrf2*^*+/+*^ hepatocytes, with FA oxidation partially rescued by treatment with AMPK activators. The unfolded protein response (UPR) was perturbed in control regular-chow (RC)-fed *Nrf2*^−/−^ mouse livers, and this was associated with constitutive activation of NF-κB and JNK, along with upregulation of inflammatory genes. The HF diet elicited an antioxidant response in *Nrf2*^*+/+*^ livers, and as this was compromised in *Nrf2*^−/−^ livers, they suffered oxidative stress. Therefore, Nrf2 protects against NASH by suppressing lipogenesis, supporting mitochondrial function, increasing the threshold for the UPR and inflammation, and enabling adaptation to HF-diet-induced oxidative stress.

## INTRODUCTION

Nonalcoholic fatty liver disease (NAFLD) is a common condition that is associated with obesity, type 2 diabetes mellitus, and insulin resistance (1, 2). It represents a spectrum of phenotypes ranging from simple steatosis (fatty infiltration) through nonalcoholic steatohepatitis (NASH) to fibrosis and ultimately cirrhosis. However, the molecular events that dictate the evolution of NASH are not well defined (3).

Consumption of a high-fat (HF) diet, or excess lipogenesis, can lead to endoplasmic reticulum (ER) stress, which in turn stimulates the unfolded protein response (UPR) that attenuates transcriptional and translational programs to restore ER homeostasis (4–6). Initiation of the UPR is carried out by activating transcription factor 6 (ATF6), inositol-requiring enzyme 1 (IRE1), and protein kinase RNA (PKR)-like ER kinase (PERK). If ER stress is unresolved, ATF6, IRE1, and PERK stimulate lipogenesis by activating sterol regulatory element binding protein 1c (SREBP-1c), X-box binding protein 1 (XBP1), CCAAT/enhancer-binding protein β (C/EBPβ), and eukaryotic initiation factor 2α (eIF2α) (7–10). Steatosis can arise during unresolved ER stress as a consequence of upregulation of C/EBP homologous protein (CHOP) because it suppresses the expression of key metabolic gene regulators (11, 12).

Progression of liver steatosis to NASH requires the coexistence of inflammation. NASH may develop in individuals with metabolic syndrome because excessive delivery of fatty acids (FA) and triglycerides to the liver leads to increased lipid metabolism, causing oxidative stress through overproduction of reactive oxygen species (ROS) (13). Oxidative stress activates nuclear factor κB (NF-κB) and c-Jun N-terminal kinase (JNK), leading to induction of inflammatory genes, which in turn increase neutrophil recruitment to the liver and exacerbate both oxidative stress and inflammation (14). Besides oxidative stress, NF-κB and JNK can also be activated by ER stress (15, 16), and the fact that ER stress is accentuated by ROS (17) indicates that inflammation in NAFLD is probably inextricably connected with both ER and oxidative stress. Once established, chronic inflammation results in induction of apoptotic genes via JNK/c-Jun signaling (14), which leads to hepatocyte death and fibrosis (18).

The cap 'n' collar (CNC) basic-region leucine zipper (bZIP) transcription factor NF-E2 p45-related factor 2 (Nrf2, also called Nfe2l2) enables cells to adapt to oxidative stress by transactivating cytoprotective genes that contain antioxidant response element (ARE) sequences in their promoters (19–21). Under nonstressed conditions, Nrf2 activity is restricted by its constitutive proteasomal degradation, which is mediated by Kelch-like ECH-associated protein 1 (Keap1), a cullin-3 ubiquitin ligase substrate adaptor (22–24). However, the ability of Keap1 to direct Nrf2 for proteasomal degradation is blocked by thiol-reactive agents, which modify Cys residues in the substrate adaptor (25–27), resulting in induction of ARE-driven genes that provide protection against electrophiles and prooxidants (28).

Nrf2 may play a pivotal role in the development of NASH, because it represses the expression of genes involved in FA synthesis (29–31) and antagonizes inflammation (32). Global knockout of Nrf2 profoundly increases the susceptibility of mice to NASH when they are placed on a methionine- and choline-deficient (MCD) diet (33, 34), whereas genetic activation of Nrf2 by knockdown of Keap1 decreases their sensitivity to NASH caused by the MCD diet (35). The MCD diet, however, is of limited value as a model for human disease because it affects only the liver, produces rapid weight loss, and does not cause insulin resistance, and therefore, challenge with an HF diet provides a more relevant means of producing NAFLD experimentally. It is therefore notable that Nrf2 has been shown to influence hepatic lipid metabolism in HF-fed mice (36–44). Although the duration of HF diet feeding has varied enormously in such studies, it has generally been found that the livers of *Nrf2*^−/−^ mice accumulate lipid to a greater extent than those of wild-type mice. Some researchers have reported greater expression of lipid metabolism-associated transcription factors and lipid metabolism-associated enzymes/proteins in *Nrf2*^−/−^ mice than in the wild type, but consistent changes in gene expression have not been observed across all studies. Furthermore, in accordance with the view that Nrf2 inhibits hepatic lipid accumulation, pharmacological activation of Nrf2 has been found to downregulate lipogenesis genes (37, 38, 45). Surprisingly, however, genetic activation of Nrf2 has been reported to increase steatosis and inflammation in both leptin-deficient and HF-fed animals (46, 47), and it has been speculated that Keap1 may influence NASH by uncharacterized mechanisms that do not involve Nrf2. Consistent with this hypothesis, evidence has been provided that Keap1 suppresses activation of the JNK/c-Jun pathway by FA in an Nrf2-independent manner (48).

The primary emphasis of all the HF diet studies in *Nrf2*^−/−^ mice outlined above has been on the expression of lipid metabolism genes. Little is therefore known about how loss of Nrf2 affects HF diet-stimulated ER stress and oxidative stress or how it influences the metabolic activity of hepatocytes. Moreover, the possible contribution of Nrf2 to insulin resistance during development of NASH is unknown. Thus, during the present study, we examined whether knockout of Nrf2 produces peripheral insulin resistance and whether this predicates increased sensitivity of the liver to NASH upon feeding an HF diet. Moreover, we tested the hypothesis that steatosis in livers of *Nrf2*^−/−^ mice fed an HF diet disturbs the UPR and causes oxidative stress, both of which drive inflammation.

## MATERIALS AND METHODS

### Animals.

Throughout this study, male mice were examined. The *Nrf2*^−/−^ and *Nrf2*^*+/+*^ animals, created by Itoh et al. (49) and provided kindly by Ken Itoh and Masayuki Yamamoto, were backcrossed over six generations onto a C57BL/6 background as described previously (50). All animal care protocols and procedures were performed in accordance to the Animal Scientific Procedures Act (1986) and with the approval of the University of Dundee Animal Ethics Committee. From 8 to 10 weeks of age, the mice were provided *ad libitum* either regular chow (RC), purchased from SDS Ltd. (Witham, Essex, United Kingdom), or an HF diet, obtained from Testdiets (International Product Supplies, London, United Kingdom). The RC contained 7.5% fat by energy; the HF diet contained 45% fat by energy. Fat mass, spontaneous locomotor activity, and food intake were determined (typically on mice between 24 and 30 weeks of age, which had been fed from the age of 8 to 10 weeks on either the HF diet or RC diet for 16 or 20 weeks) as described previously (51). For analysis of insulin signaling (*ex vivo*), mice (typically 28 weeks of age) were subjected to an overnight fast and injected with 2 units of insulin/kg body weight or an equal volume of saline intraperitoneally. The quadriceps muscle and liver were collected in liquid nitrogen 5 and 6 min after injection, respectively.

The vast majority of biochemical and molecular biology analyses were performed on livers of *Nrf2*^−/−^ and *Nrf2*^*+/+*^ mice that had been placed on an RC or HF diet for 24 weeks (32 to 34 weeks of age). Upon sacrifice of these animals, plasma was collected, and the livers were removed. A lobe from each liver was preserved in formalin for histological analyses, and the remainder was snap-frozen in liquid nitrogen.

### Physiological and clinical-chemistry measurements.

The EchoMRI-900 quantitative nuclear magnetic resonance (qMR) system (Echo Medical Systems, Houston, TX) was used to determine fat mass and lean mass in conscious mice. Blood samples were collected via tail vein or cardiac puncture performed on terminally anesthetized mice. Blood glucose, triglycerides, cholesterol and free fatty acid, and plasma leptin and insulin were measured as described previously (51). Plasma β-hydroxybutyrate was measured using a colorimetric assay (Cayman Chemical Company, Ann Arbor, MI). Plasma alanine aminotransferase (ALT) activity was measured using kits on a Daytona autoanalyzer (Randox). Glucose and insulin tolerance tests were carried out on mice as described elsewhere (51). The respiratory exchange ratio (RER) and O_2_ consumption were determined by open-circuit indirect calorimetry (Columbus Instruments).

### Histology.

Formalin-fixed murine liver specimens were processed for hematoxylin and eosin staining as described previously (33). The severity of liver disease was evaluated histologically using the NAFLD activity score (NAS), which is the standard system for reporting the extent of damage (52); it represents the combined semiquantitated pathology score for steatosis, inflammation, and hepatocyte ballooning.

Reticulin and Van Gieson's staining of liver sections was undertaken by standard methods. Staining for nitrotyrosine protein adducts was performed as described previously (53), and a terminal deoxynucleotidyl transferase-mediated dUTP-biotin nick end labeling (TUNEL) assay was performed as described elsewhere (54). For electron microscopy analysis, livers were fixed in 2.5% glutaraldehyde-4% paraformaldehyde in 0.1 M sodium cacodylate buffer, postfixed in 1% aqueous osmium tetroxide, dehydrated in ethanol, transferred to propylene oxide, and then embedded in Durcupan resin (Sigma). Sections were cut on a Leica ultramicrotome and collected on Pioloform B (polyvinyl butyral)-coated copper grids, stained with uranyl acetate and lead citrate, and examined in a Jeol EX electron microscope. Images were collected on digital-imaging plates and processed in a Ditabis (Pforzeim, Germany) scanner.

### Antibodies.

Antibodies against acetyl coenzyme A (CoA) carboxylase (ACC), phosphorylated ACC (p-ACC), phosphorylated AMP-activated protein kinase (p-AMPK), CHOP, cleaved caspase 3, cleaved caspase 9, eIFα, 78-kDa glucose-regulated protein (GRP78, also called BiP), high-mobility group protein B1 (HMGB1), IRE1α, PERK, JNK, and p-JNK were purchased from Cell Signaling (Invitrogen). The antibodies against activating transcription factor 4 (ATF4), pHistone H2AX, NF-κB, and procaspase 9 were obtained from Santa Cruz. The antibody against ATF6 was from Imgenex, and that against XBP1 was from Abcam. The antibody against actin was from Sigma-Aldrich, and that against total AMPK (α1 and α2 subunits) was from DSTT (University of Dundee). The antibody against nitrotyrosine was from Millipore. Antisera against mouse glutathione *S*-transferase a1 (Gsta1), mouse Gstm1, rat GSTA4, human glutamate-cysteine ligase catalytic (GCLC) and modifier (GCLM) subunits, and rat NAD(P)H:quinone oxidoreductase 1 (NQO1) were produced in house (50, 55).

### Biochemical analyses.

Portions (approximately 100 mg) of frozen mouse liver were pulverized individually under liquid nitrogen, using a mortar and pestle. The ground material from each sample was resuspended in 1 ml of ice-cold 50 mM HEPES buffer, pH 7.5, that contained 150 mM NaCl, 1 mM dithiothreitol, and protease inhibitors before being homogenized. Thereafter, cytosol was prepared at 4°C from individual livers after two centrifugation steps (15,000 × *g* for 45 min and 100,000 × *g* for 90 min). For AMPK, ACC, and JNK Western blots, whole-cell soluble extracts were prepared from the resuspended material by a single centrifugation step (15,000 × *g* for 15 min at 4°C). For the NF-κB Western blots, nuclear extracts were prepared from frozen liver using the Pierce NE-PER kit (ThermoScientific Life Science Research Products, Rockford, IL). Protein concentrations in samples were measured as described previously, as were total glutathione, reduced glutathione (GSH), and oxidized glutathione (GSSG) (33). Liver malondialdehyde (MDA) and protein oxidation levels were measured using a TBARS assay kit (Cayman Chemical Company) and an OxyBlot detection kit (Millipore), respectively.

### Gene expression profiling.

The expression of hepatic genes in the *Nrf2*^−/−^ and *Nrf2*^*+/+*^ mice fed an RC or HF diet was measured by TaqMan real-time PCR using an Applied Biosystems Prism model 7700 sequence detector instrument (50). mRNAs for the following classes of proteins were monitored using commercial primer and probe sets, all of which were purchased from Life Technologies: lipid-associated transcription factors (farnesoid X receptor [FXR; Mm00436425_m1], liver X receptor alpha [LXRα; Mm00443451_m1], LXRβ [Mm00437265_g1], MLX-interacting protein-like [Mlxipl, also called carbohydrate-responsive element binding protein {ChERBP}; Mm00498811_m1], peroxisome proliferator-activated receptor alpha [PPARα; Mm00440939_m1], PPARγ [Mm01184322_m1], retinoid X receptor alpha [RXRα; Mm00441185_m1], small heterodimer partner [Shp; Mm00442278_m1], Srebf1 [whose mRNA encodes Srebp-1c; Mm00550338_m1], and Srebf2 [whose mRNA encodes Srebp-2; Mm01306293_m1]), FA synthesis proteins (acetyl-CoA carboxylase alpha [Acaca; Mm01304277_m1], ATP-citrate lyase [Acly; Mm01302282_m1], elongation of very long-chain fatty acids protein 5 [Elovl5; Mm00506717_m1], Elovl6 [Mm00851223_s1], and fatty acid synthase [Fasn; Mm00662319_m1]), the FA desaturation enzyme stearoyl-CoA desaturase (Scd1; Mm00772290_m1), triglyceride assembly proteins (fatty acid-binding protein 1 [Fabp1; Mm00444340_m1] and Fabp5 [Mm00783731_s1]), enzymes involved in FA oxidation (acetyl-CoA acyltransferase 1 [Acaa1; Mm00728460_s1], acetyl-CoA carboxylase beta [Acacb; Mm01204683_ml], carnitine palmitoyl-transferase [Cpt1a; Mm00550439_m1], cytochrome P450 4a10 [Cyp4a10; Mm01188913_g1], Cyp4a14 [Mm00484132_m1], and Cyp2e1 [Mm00491127_m1]), ER stress proteins (Atf4 [Mm00515325_g1], Atf6 [Mm00520279_m1], Chop [Mm01135937_g1], growth arrest and DNA-damage-inducible protein 34 [Gadd34; Mm01205601_g1], Grp78 [Mm00517690_g1], and Xbp1s and Xbp1unspliced [56]), inflammation proteins (cyclooxygenase 2 [Cox2; Mm00478377_g1], interleukin-1β [IL-1β; Mm99999061_mH], IL-6 [Mmoo446190_m1], myeloperoxidase [Mpo; Mm01298424_m1], nitric oxide synthase 2 [Nos2; Mm00440485_m1], NF-κB p65 [RelA; Mm00501346_m1], and tumor necrosis factor alpha [TNF-α; Mm00443259_g1]), antioxidant proteins (cystine/glutamate antiporter light chain xCT [Slc7a11; Mm00442530_m1], Gclc [50], Gclm [50], sulfiredoxin [Srxn1; Mm00769566_m1], and thioredoxin reductase [Txnrd1; Mm00443675_m1]), drug-metabolizing enzymes (Gsta1 [50], Gsta2 [50], Gsta4 [50], Gstm1 [50], and Gstm2 [50] subunits and Nqo1 [57]), and the general stress enzyme heme oxygenase 1 (Hmox1; Mm00516006_m1).

### Western blotting.

Tissue proteins were resolved by SDS-PAGE in 10% polyacrylamide gels before they were transferred onto Immobilon-P membranes. The immobilized polypeptides were probed using a variety of commercial antibodies or in-house rabbit antiserum, and following extensive washing of the blots, the cross-reacting bands were identified using horseradish peroxidase (HRP)-conjugated goat anti-rabbit IgG and visualized by enhanced chemiluminescence (50, 55). Equal protein loading of samples was assessed using glyceraldehyde-3-phosphate dehydrogenase (GAPDH), actin, or PCNA.

### Cells.

Primary hepatocytes were prepared from the livers of RC diet-fed 10-week-old mice by the method of Foretz et al. (58). The livers were first washed with perfusion buffer (137 mM NaCl, 7 mM KCl, 0.7 mM Na_2_HPO_4_, and 10 mM HEPES, adjusted to pH 7.65) containing 0.5 mM EDTA to remove blood before they were treated with 400 μg/ml collagenase in the perfusion buffer. Following isolation, primary hepatocytes were plated in M199 medium plus GlutaMax (Invitrogen) supplemented with 10% fetal calf serum (FCS), 1% penicillin-streptomycin, 0.1% bovine serum albumin (BSA), 10 nM insulin, 200 nM triiodothyronine, and 500 nM dexamethasone.

### Metabolic studies.

Experiments were performed to determine whether the rates of mitochondrial respiration or glycolysis differed in *Nrf2*^−/−^ and *Nrf2*^*+/+*^ hepatocytes upon treatment with fatty acids or glucose using a Seahorse Bioscience XF 24 analyzer. Freshly prepared primary hepatocytes were plated in XF 24-well plates at a density of 1 × 10^4^ cells per well in M199 medium containing 2 mM GlutaMax, which was supplemented with 10% FCS, 1% penicillin-streptomycin, 0.1% BSA, 10 nM insulin, 200 nM triiodothyronine, and 500 nM dexamethasone. The cells were cultured overnight at 37°C before being washed with XF 24 Dulbecco's modified Eagle's medium (DMEM) assay medium supplemented with 5.5 mM glucose and 2 mM GlutaMax prior to incubation for 1 h in the same medium (without CO_2_ preincubation). Thereafter, the plates were loaded onto an XF 24 analyzer to measure the oxygen consumption rate (OCR) at regular intervals as cells alone (baseline) and after injecting BSA conjugated with 375 μM palmitic acid or 375 μM oleic acid in the presence of 1 mM carnitine. The values were normalized against the total protein content of the cells in each well.

### Statistics.

Comparisons between the biochemical and molecular biology results from the four experimental groups were made using a paired or unpaired two-tailed Student's *t* test, a one-sample Student's *t* test, or analysis of variance (ANOVA) with a Bonferroni *post hoc* analysis as appropriate. Analysis of covariance (ANCOVA) was used to examine the relationship between body weight and oxygen consumption. The results are means and standard errors of the mean (SEM), and *P* values of ≤0.05 were considered statistically significant. Comparisons between the histology NAS results were made using the Kruskal-Wallis H test.

## RESULTS

### Whole-body metabolic response to a high-fat diet is modified by Nrf2.

**FIG 1:**
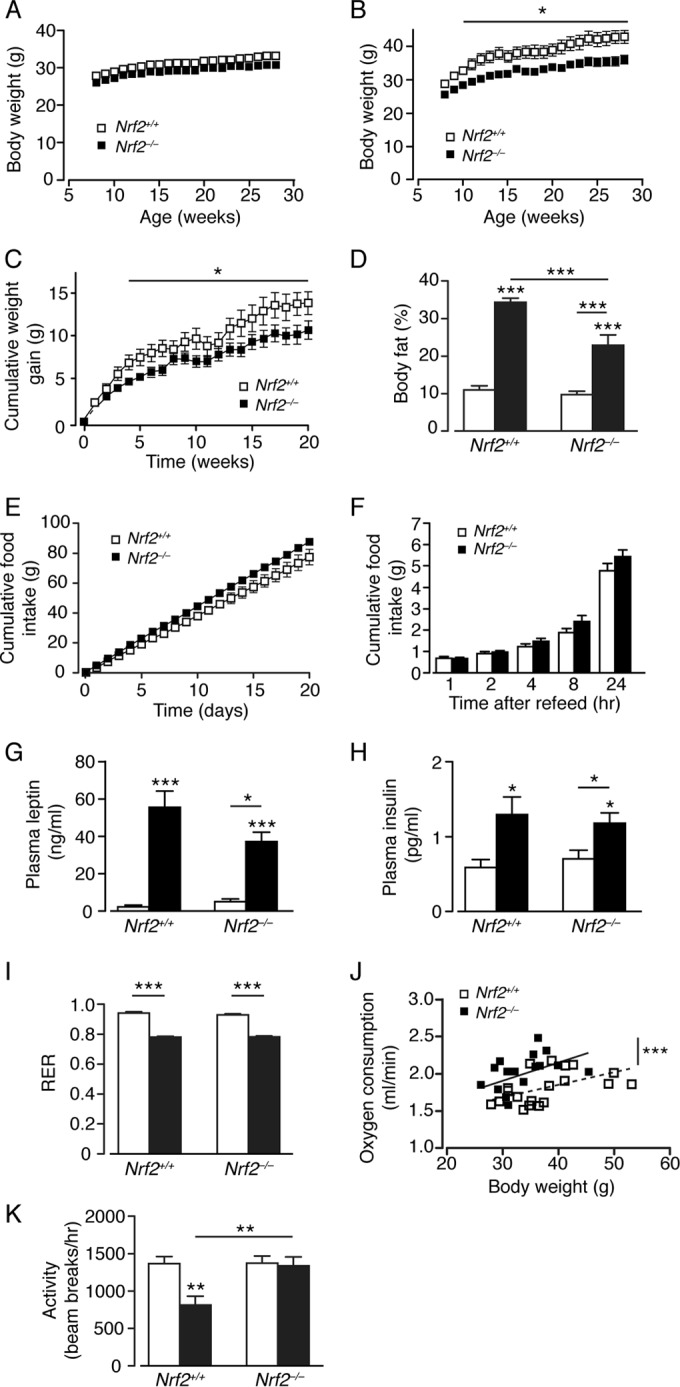
*Nrf2*^−/−^ mice on an HF diet exhibit reduced adiposity and higher energy expenditure than *Nrf2*^*+/+*^ mice. (A and B) Body mass curves of male age-matched *Nrf2*^*+/+*^ and *Nrf2*^−/−^ mice fed on an RC (A) or HF (B) diet for 20 weeks from 8 weeks of age (10 or 11 mice per group). (C) Body mass gains of *Nrf2*^*+/+*^ and *Nrf2*^−/−^ mice fed on an HF diet. (D) Percentages of body fat in *Nrf2*^*+/+*^ and *Nrf2*^−/−^ mice on an RC or HF diet (12 or 13 mice per group). (E) Cumulative food intake of age-matched male *Nrf2*^*+/+*^ and *Nrf2*^−/−^ mice fed on RC over a period of 20 weeks (6 mice per group). (F) Food intake after overnight fast in age-matched *Nrf2*^*+/+*^ and *Nrf2*^−/−^ mice fed on an HF diet (5 mice per group). (G and H) Fed plasma leptin (G) and insulin (H) levels from *Nrf2*^*+/+*^ and *Nrf2*^−/−^ mice on an RC or HF diet (9 to 11 mice per group). (I and J) RER (I) and oxygen consumption (J) (the lines show fitted regressions) for *Nrf2*^*+/+*^ and *Nrf2*^−/−^ mice on an RC or HF diet (7 to 13 mice per group). (K) Effects of genotype and diet on locomotor activity for *Nrf2*^*+/+*^ and *Nrf2*^−/−^ mice (7 to 13 mice per group). (D, G to I, and K) White bars, RC fed; black bars, HF fed. The results are means and SEM. *, *P* < 0.05; **, *P* < 0.01; ***, *P* < 0.001.

*Nrf2*^−/−^ mice fed an RC diet starting from 8 to 10 weeks of age did not differ from wild-type mice in average length (data not shown) or body mass (Fig. 1A); *Nrf2*^−/−^ mice appeared consistently lighter, although this difference did not reach significance. When fed an HF diet, mass accumulation was less apparent in *Nrf2*^−/−^ mice (Fig. 1B and C), with a mean increase in body mass of 14.5 ± 1.3 g (*n* = 10) and 10.7 ± 1.1 g (*n* = 11; *P* < 0.05) for *Nrf2*^*+/+*^ and *Nrf2*^−/−^ mice, respectively. At 28 weeks of age, qMR scanning revealed that RC-fed *Nrf2*^−/−^ and *Nrf2*^*+/+*^ mice had identical body fat levels and that HF-fed *Nrf2*^−/−^ and *Nrf2*^*+/+*^ mice increased their relative fat mass, but significantly less for the *Nrf2*^−/−^ mice (Fig. 1D).

Daily food intake was slightly greater for RC-fed *Nrf2*^−/−^ than for *Nrf2*^*+/+*^ mice (Fig. 1E), but no difference in food consumption was observed in HF-fed *Nrf2*^−/−^ and *Nrf2*^*+/+*^ mice by determining compensatory feeding following an overnight fast (Fig. 1F). Plasma leptin and insulin levels were similar in RC-fed *Nrf2*^−/−^ and *Nrf2*^*+/+*^ mice, but consistent with their increased body fat content, HF-fed *Nrf2*^−/−^ and *Nrf2*^*+/+*^ mice displayed increased plasma leptin and insulin levels, with a nonsignificant trend for reduced leptin levels in *Nrf2*^−/−^ mice (Fig. 1G and H). No difference was observed in the respiratory exchange ratio between *Nrf2*^−/−^ and *Nrf2*^*+/+*^ mice, though it was lowered by the HF diet in both (Fig. 1I). Indirect calorimetry showed that *Nrf2*^−/−^ mice exhibit higher O_2_ consumption, indicating that loss of Nrf2 increases energy expenditure (Fig. 1J). HF feeding decreased the ambulatory activity of *Nrf2*^*+/+*^ mice in comparison to RC-fed *Nrf2*^*+/+*^ mice, whereas ambulatory activity was unchanged by diet in *Nrf2*^−/−^ mice (Fig. 1K). Thus, although *Nrf2*^−/−^ mice display sensitivity to HF feeding by increasing body fat content, the magnitude of this effect was less than was observed in *Nrf2*^*+/+*^ mice, likely due to the increased energy expenditure of *Nrf2*^−/−^ mice.

### Insulin sensitivity is maintained in *Nrf2*^−/−^ mice fed the high-fat diet.

**FIG 2:**
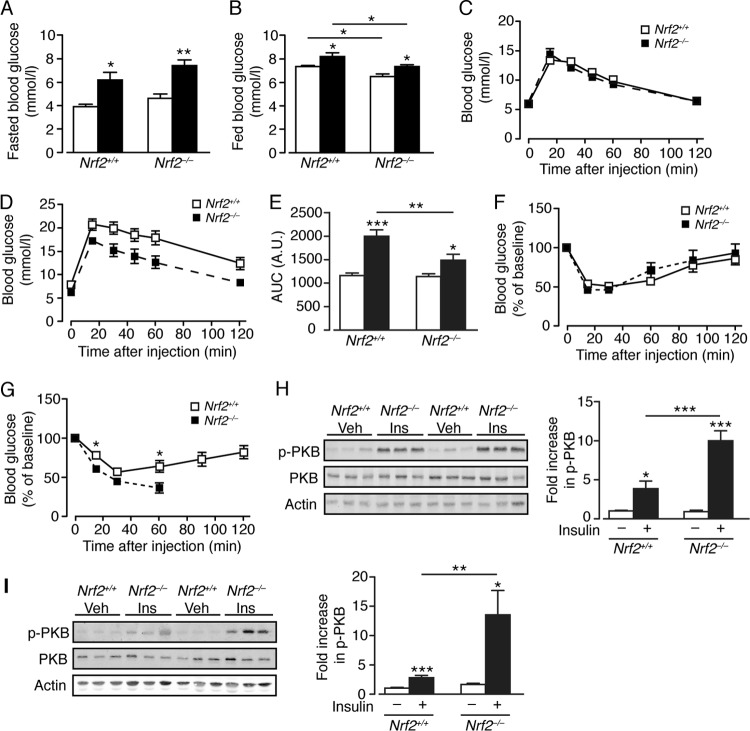
*Nrf2*^−/−^ mice display improved glucose homeostasis and insulin sensitivity with enhanced skeletal muscle and liver insulin signaling. (A and B) Fasted (A) and fed (B) blood glucose levels in *Nrf2*^*+/+*^ and *Nrf2*^−/−^ mice after 16 weeks on an RC or HF diet (5 or 6 mice per group). (C) GTT on *Nrf2*^*+/+*^ (*n* = 11) and *Nrf2*^−/−^ (*n* = 9) mice, 28 weeks old, fed RC. (D) GTT on *Nrf2*^*+/+*^ and *Nrf2*^−/−^ mice fed an HF diet for 20 weeks from 8 weeks of age (11 mice per group). (E) Quantification of the area under the curve (AUC) for the total glycemic excursions shown in panels C and D by genotype and diet. (F) ITT on *Nrf2*^*+/+*^ (*n* = 11) and *Nrf2*^−/−^ (*n* = 9) mice, 28 weeks old, fed RC. (G) ITT on *Nrf2*^*+/+*^ (*n* = 11) and *Nrf2*^−/−^ (*n* = 10) mice fed an HF diet for 20 weeks from 8 weeks of age. Note the curtailed time course of tolerance tests on *Nrf2*^−/−^ mice owing to continual decline in blood glucose and the requirement to intervene with glucose administration. (H and I) Representative immunoblots of total PKB and insulin-stimulated phosphorylation of PKB (also called Akt) at Ser^473^ (p-PKB) from the liver (H) and skeletal muscle (I) of *Nrf2*^*+/+*^ and *Nrf2*^−/−^ mice (7 months old) fed RC (10 to 19 mice per group). Quantification of the immunoblot data is shown, and in each case, p-PKB/PKB was normalized with respect to RC-fed *Nrf2*^*+/+*^ levels. White bars, RC fed; black bars, HF fed. The results are means and SEM. *, *P* < 0.05; **, *P* < 0.01; ***, *P* < 0.001.

As NASH is associated with insulin resistance, we examined glucose homeostasis. On an RC diet, fasting blood glucose levels were similar in *Nrf2*^*+/+*^ and *Nrf2*^−/−^ mice, and HF feeding for 16 weeks raised fasted glucose levels in both groups, indicative of decreased liver insulin sensitivity (Fig. 2A). Although HF feeding produced a significant increase in fed glucose levels in both *Nrf2*^−/−^ and *Nrf2*^*+/+*^ mice, fed glucose levels were lower in the mutant than in wild-type mice for both RC and HF diets (Fig. 2B). Intraperitoneal glucose tolerance tests (GTT) showed that RC-fed *Nrf2*^−/−^ and *Nrf2*^*+/+*^ mice had comparable glucose disposal (Fig. 2C and E), whereas HF-fed *Nrf2*^−/−^ mice displayed significantly better glucose disposal from the peripheral circulation than diet- and age-matched *Nrf2*^*+/+*^ mice (Fig. 2D and E). Also, HF-fed *Nrf2*^−/−^ mice exhibited a greater decrease in blood glucose during insulin tolerance tests (ITT) than *Nrf2*^*+/+*^ mice, an outcome not observed for RC-fed mice (Fig. 2F and G); importantly, the ITT for HF-fed *Nrf2*^−/−^ mice had to be curtailed after 60 min, as ∼50% of them did not mount a counterregulatory response to insulin-induced hypoglycemia and had to be given glucose to recover.

To further assess peripheral insulin signaling strength in *Nrf2*^−/−^ and *Nrf2*^*+/+*^ mice, we measured the levels of protein kinase B phosphorylated at Ser473 (p-PKB; also called Akt) in liver and skeletal muscle following intraperitoneal injection of insulin. In nonstimulated tissues, p-PKB levels did not differ in the two groups of mice. However, in response to insulin, *Nrf2*^−/−^ mice displayed significantly higher p-PKB levels in liver and skeletal muscle than *Nrf2*^*+/+*^ mice (Fig. 2H and I). Therefore, *Nrf2*^−/−^ mice exhibit greater peripheral insulin sensitivity than *Nrf2*^*+/+*^ mice, and consequently, when challenged chronically with the HF diet, *Nrf2*^−/−^ mice retain better insulin-mediated glucose disposal than HF-fed *Nrf2*^*+/+*^ mice.

### Nrf2^−/−^ mice develop a NASH phenotype when fed a high-fat diet.

**FIG 3:**
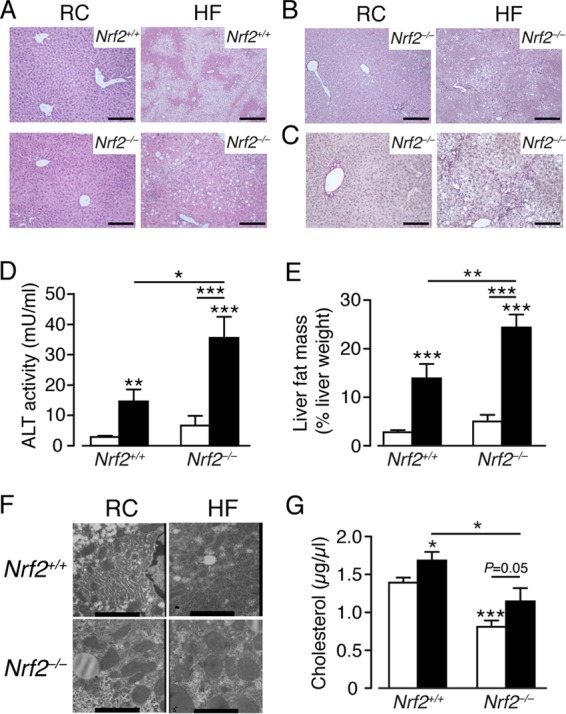
Histological and biochemical evidence of steatohepatitis and fibrosis in the livers of *Nrf2*^−/−^ mice fed a high-fat diet. (A) Representative H&E-stained images of livers from *Nrf2*^*+/+*^ and *Nrf2*^−/−^ mice after 24 weeks on an RC or HF diet from 8 to 10 weeks of age. (B and C) Representative images of reticulin staining (B) and Van Gieson's staining (C) in livers from *Nrf2*^−/−^ mice after 24 weeks on an RC or HF diet. Bars, 100 μm. (D) ALT activity in plasma of *Nrf2*^*+/+*^ and *Nrf2*^−/−^ mice after 24 weeks on an RC or HF diet (8 to 11 mice per group). (E) Magnetic resonance analysis of fat content in livers from *Nrf2*^*+/+*^ and *Nrf2*^−/−^ mice on an RC or HF diet (12 or 13 mice per group). (F) Representative electron microscope images of liver sections from *Nrf2*^*+/+*^ and *Nrf2*^−/−^ mice on an RC or HF diet; bars, 1,731 nm. (G) Cholesterol levels in plasma from *Nrf2*^*+/+*^ and *Nrf2*^−/−^ mice on RC and HF diets (9 to 11 mice per group). (D, E, and G) White bars, RC fed; black bars, HF fed. The results are means and SEM. *, *P* < 0.05; **, *P* < 0.01; ***, *P* < 0.001.

Hematoxylin and eosin (H&E) staining of liver sections was assessed by an expert histopathologist, using the NAS scoring system developed for human disease. Individual scores for fat, inflammation, and fibrosis were also analyzed. Livers from RC-fed *Nrf2*^*+/+*^ mice showed normal hepatocyte architecture and no evidence of steatosis. However, livers of HF-fed *Nrf2*^*+/+*^ mice developed hepatic steatosis without significant histological evidence of either inflammation or fibrosis that is consistent with simple steatosis of the human NAFLD spectrum but not with NASH (Fig. 3A and Table 1). Livers from RC-fed *Nrf2*^−/−^ mice displayed signs of microvesicular steatosis but no inflammation. However, livers from HF-fed *Nrf2*^−/−^ mice showed significant microvesicular and macrovesicular steatosis, neutrophil infiltration, apoptotic bodies, and disruption of hepatic architecture that are consistent with NASH. Cirrhosis was observed only in livers of HF-fed *Nrf2*^−/−^ mice. In conclusion, HF-fed *Nrf2*^*+/+*^ and HF-fed *Nrf2*^−/−^ livers contained similar levels of fat that were significantly higher than in RC-fed *Nrf2*^−/−^ livers, and all contained more fat than RC-fed *Nrf2*^*+/+*^ livers. Moreover, the HF-fed *Nrf2*^−/−^ livers had significantly more inflammation than any other group. All of these observations are based upon scores that are relative to each other and to the normal, rather than absolute values.

**TABLE 1:** Histology of livers from *Nrf2^+/+^* and *Nrf2*^−/−^ mice fed a high-fat diet for 24 weeks

Parameter	Value^*f*^ (*n*)	
	RC diet	HF diet
NAS^*a*^ (maximum 8)		
Nrf2^+/+^ mice	0.5 (10)	3.45 (11)
Nrf2^−/−^ mice	2.0 (9)	4.9 (10)
Steatosis component of NAS^*b*^ (0–3)		
Nrf2^+/+^ mice	0.9 (10)	2.55 (11)
Nrf2^−/−^ mice	1.33 (9)	2.4 (10)
Inflammatory component of NAS^*c*^ (0–3)		
Nrf2^+/+^ mice	0 (10)	0.82 (11)
Nrf2^−/−^ mice	0.66 (9)	2.3 (10)
Ballooning component of NAS^*d*^ (0–2)		
Nrf2^+/+^ mice	0 (10)	0 (11)
Nrf2^−/−^ mice	0 (9)	0.3 (10)
Fibrosis stage^*e*^ (0–3)		
Nrf2^+/+^ mice	0 (10)	0 (11)
Nrf2^−/−^ mice	0 (9)	1 (10)

a In *Nrf2^+/+^* mice, the NAS is significantly higher in livers of HF-fed mice than in RC-fed animals (Kruskal-Wallis H test; *P* = 0.002), and the same is seen in livers of HF-fed *Nrf2*^−/−^ mice compared to RC-fed *Nrf2*^−/−^ livers. The HF-fed *Nrf2*^−/−^ mice had a significantly higher NAS than HF-fed *Nrf2^+/+^* mice (Kruskal-Wallis H test; *P* = 0.03).

b Both HF-fed *Nrf2^+/+^* and *Nrf2*^−/−^ mice had more steatosis than their RC-fed counterparts (Kruskal-Wallis H test; *P* = 0.03). No significant difference was observed between *Nrf2^+/+^* and *Nrf2*^−/−^ mice fed the same diet.

c The inflammatory component of the NAS was significantly higher in livers of HF-fed *Nrf2*^−/−^ mice than in either HF-fed *Nrf2^+/+^* or RC-fed *Nrf2*^−/−^ mice (Kruskal-Wallis H test; *P* = 0.002).

d No statistical differences were observed for ballooning between the experimental groups.

e The median values are shown, and fibrosis was observed only in livers of HF-fed *Nrf2*^−/−^ mice.

f The significance of differences in the NAS scores of livers from RC-fed and HF-fed *Nrf2^+/+^* and *Nrf2*^−/−^ mice was assessed using the Kruskal-Wallis H test. The differences between the NAS results of the 4 experimental groups were highly significant (Kruskal-Wallis H test; *P* = 0.002), with both HF diet mice having higher NAS values than their chow-fed counterparts. Moreover, a significant difference was observed between the two HF-fed groups, with the *Nrf2*^−/−^ animals having the higher score (Kruskal-Wallis H test; *P* = 0.03), despite the fact that the steatosis components of NAS were similar in the two HF-fed groups, which blurs the difference in their NAS results. The difference in the NAS between HF-fed *Nrf2^+/+^* and *Nrf2*^−/−^ animals is smaller than the difference in inflammation and the degree of NASH between them; this represents a limitation of the NAS ranking system, which simply adds the 3 components together. Therefore, the apparent relative increase of steatosis in HF-fed *Nrf2^+/+^* livers compared to HF-fed *Nrf2*^−/−^ livers masks the increased inflammation score in the latter, because hepatocytes lose fat as they become inflamed. Fibrosis, which is not part of the NAS, was not observed in the livers of RC-fed animals or in HF-fed *Nrf2^+/+^* livers, but it was seen in HF-fed *Nrf2*^−/−^ livers (amounting to cirrhosis in 2 of 10 animals).

Reticulin staining revealed that HF-fed *Nrf2*^*+/+*^ livers contained significantly increased amounts of type III collagen compared with their RC-fed counterparts and that RC-fed *Nrf2*^−/−^ mouse livers had levels of type III collagen similar to those of *Nrf2*^*+/+*^ mice on the same diet (data not shown). However, livers from HF-fed *Nrf2*^−/−^ mice contained larger amounts of collagen than those of HF-fed *Nrf2*^*+/+*^ mice (Fig. 3B). Van Gieson's staining of hepatic sections revealed normal levels of collagen in RC-fed *Nrf2*^−/−^ livers but large increases in collagen deposition in HF-fed *Nrf2*^−/−^ livers from the central vein through hepatocytes to the portal triad (Fig. 3C). Increased liver pathology in HF-fed *Nrf2*^−/−^ mice was accompanied by elevated plasma ALT activity (Fig. 3D).

qMR analysis revealed that RC-fed *Nrf2*^−/−^ and RC-fed *Nrf2*^*+/+*^ livers had comparable levels of fat and that HF feeding increased fat deposition in both genotypes, with *Nrf2*^−/−^ livers containing the most fat among the four groups (Fig. 3E). Ultrastructural analysis showed normal architecture for RC-fed *Nrf2*^*+/+*^ livers, with fat globules present in hepatocytes from HF-fed *Nrf2*^*+/+*^ and RC-fed *Nrf2*^−/−^ mice, whereas hepatocytes of HF-fed *Nrf2*^−/−^ mice were characterized by swollen mitochondria with reduced crista and disrupted membranes (Fig. 3F). HF feeding did not increase plasma triglycerides or FA levels in either genotype (data not shown) but increased plasma cholesterol in both genotypes; *Nrf2*^−/−^ mice exhibited lower cholesterol on either diet than *Nrf2*^*+/+*^ mice (Fig. 3G), and this is most likely due to inactivation of the redox-sensitive HMG CoA reductase in the mutant mouse by higher ROS levels (59).

Overall, a clear progression was seen in the animal models across the NAFLD disease spectrum, with RC-fed *Nrf2*^*+/+*^ mice representing normality; RC-fed *Nrf2*^−/−^ mice and HF-fed *Nrf2*^*+/+*^ mice developing steatosis, worse in the latter; and HF-fed *Nrf2*^−/−^ mice developing the complete NASH phenotype with fibrosis. We therefore sought, in the experiments described below, to determine molecular changes that account for the greater degree of NAFLD, inflammation, hepatocellular death, and oxidative stress in mice lacking Nrf2.

### Induction of lipogenesis genes by a high-fat diet is accentuated in *Nrf2*^−/−^ livers.

**FIG 4:**
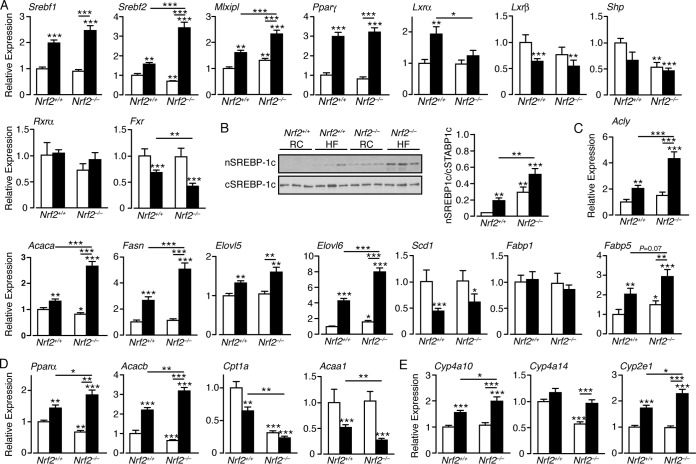
Expression of many lipid-associated transcription factors and lipogenic enzymes is upregulated in HF-fed *Nrf2*^−/−^ livers to a greater extent than in HF-fed *Nrf2*^*+/+*^ livers. (A) Quantitative-PCR analysis of mRNAs for transcription factors that regulate lipid and glucose metabolism and storage in livers of *Nrf2*^*+/+*^ and *Nrf2*^−/−^ mice on an RC or HF diet (6 to 12 mice per group). (B) Immunoblots of Srebp-1c in nuclear subcellular fractions (nSREBP-1c) and cytosolic subcellular fractions (cSREBP-1c) from livers of *Nrf2*^*+/+*^ and *Nrf2*^−/−^ mice on an RC or HF diet. (C and D) Quantitative-PCR analysis of mRNA for enzymes involved in lipogenesis (C) and FA catabolism (D) in livers of *Nrf2*^*+/+*^ and *Nrf2*^−/−^ mice on an RC or HF diet (10 to 12 mice per group). (E) Quantitative-PCR analysis of mRNA for cytochrome P450 enzymes that oxidize fatty acids in livers of *Nrf2*^*+/+*^ and *Nrf2*^−/−^ mice on an RC or HF diet (10 to 12 mice per group). In each case, the data were normalized with respect to RC-fed *Nrf2*^*+/+*^ levels for each gene. White bars, RC fed; black bars, HF fed. The results are means and SEM. *, *P* < 0.05; **, *P* < 0.01; ***, *P* < 0.001.

First, we examined the mRNA levels of the following transcription factors that contribute to liver steatosis: Srebf1 and Srebf2, which regulate cholesterol biosynthesis; Mlxipl, which regulates triglyceride synthesis genes in a glucose-dependent manner; PPARγ, which promotes storage of FA; LXRα and LXRβ, which control lipid homeostasis and inflammation; Frx, which regulates cholesterol and bile acid production; and Shp, which represses LXR (for reviews, see references 60 to 64). In *Nrf2*^*+/+*^ livers, the HF diet increased mRNA levels for Srebf1, Srebf2, Mlxip, PPARγ, and LXRα but had no significant effect on mRNA for Shp and reduced mRNAs for LXRβ and FXR (Fig. 4A). Loss of Nrf2 had a moderate impact on liver expression of these transcription factors, with a modest increase in Mlxipl and more significant decreases in mRNAs for Srebf2 and Shp. However, HF-fed *Nrf2*^−/−^ livers exhibited significant increases in mRNAs for Srebf1, Srebf2, Mlxipl, and PPARγ, with no change in mRNA for LXRα and reduced levels of mRNAs for Shp, LXRβ, and FXR (Fig. 4A). Western blotting revealed that the active form of Srebp-1 (encoded by *Srebf1*) was most abundant in the nuclei of HF-fed *Nrf2*^−/−^ livers but was also elevated in the nuclei of RC-fed *Nrf2*^−/−^ and HF-fed *Nrf2*^*+/+*^ livers compared with RC-fed *Nrf2*^*+/+*^ livers (Fig. 4B). Thus, the HF diet increased expression in the liver of transcription factors that are associated with FA uptake, FA oxidation, FA storage, lipogenesis, and inflammation in wild-type and *Nrf2*^−/−^ mice, but in all cases, the effect was greater in *Nrf2*^−/−^ than in *Nrf2*^*+/+*^ livers. Also, the HF diet decreased expression of FXR, which controls the synthesis of bile acids, as well as the expression of the nuclear receptor repressor Shp, which is regulated by *Fxr* (60), and again, this was most apparent in *Nrf2*^−/−^ livers.

Second, mRNAs for proteins involved in lipogenesis were examined. They included Acly, which catalyzes production of acetyl-CoA that is required for lipid biosynthesis; the FA synthesis *Acaca* gene, which encodes AccI that produces malonyl-CoA; the FA synthase Fasn; the FA elongases Elovl5 and Elovl6; the desaturase Scd1; and the FA uptake and transport proteins Fabp1 and Fabp5 (61, 64). In *Nrf2*^*+/+*^ mice, HF feeding increased mRNAs for Acly, Acaca, Elovl5, Elovl6, Fasn, and Fabp5 but had no effect on mRNA for Fabp1 and reduced mRNA for Scd1 (Fig. 4C). Livers from RC-fed *Nrf2*^−/−^ mice showed a modest decrease in mRNA for Acaca and an increase in Elovl6 and Fabp5, with no change in the other transcripts. However, HF-fed *Nrf2*^−/−^ livers demonstrated increases in mRNAs for Acly, Acaca, Fasn, Elovl5, Elovl6, and Fabp5 that were significantly greater than those observed in HF-fed *Nrf2*^*+/+*^ livers but had no effect on Fabp1. The HF diet appeared to downregulate mRNA for *Scd1* expression independently of genotype (Fig. 4C). Thus, while the HF diet increased the expression of several key enzymes/proteins associated with lipogenesis, this induction was accentuated in *Nrf2*^−/−^ livers.

### Lipid catabolism genes are downregulated in *Nrf2*^−/−^ livers.

Expression of *Ppar*α, which supports uptake, utilization, and catabolism of FA, was increased in HF-fed *Nrf2*^*+/+*^ livers (Fig. 4D). The expression of *Ppar*α in RC-fed *Nrf2*^−/−^ livers was lower than in wild-type controls. However, induction of *Ppar*α in HF-fed *Nrf2*^−/−^ livers exceeded that seen in wild-type mice. Similarly, expression of the *Acacb* gene, which encodes Acc2 and results in raised malonyl-CoA, causing inhibition of Cpt1a, the rate-limiting step in FA uptake into mitochondria, was increased by the HF diet in *Nrf2*^*+/+*^ livers and to a much greater extent in HF-fed *Nrf2*^−/−^ livers. In addition, *Acacb* gene expression was lower in RC-fed *Nrf2*^−/−^ livers than in RC-fed *Nrf2*^*+/+*^ livers.

RC- and HF-fed *Nrf2*^−/−^ livers had much reduced mRNA levels for Cpt1a, which is required for β-oxidation of long-chain FA, compared to RC-fed *Nrf2*^*+/+*^ livers, with the HF diet reducing Cpt1a mRNA in *Nrf2*^*+/+*^ livers to a much lesser extent than in *Nrf2*^−/−^ livers (Fig. 4D). The expression of *Acaa1*, which contributes to the β-oxidation of FA in peroxisomes, was diminished in HF-fed *Nrf2*^*+/+*^ livers, but it was further lowered in HF-fed *Nrf2*^−/−^ livers. Thus, the increased expression of *Acacb* in HF-fed *Nrf2*^−/−^ livers, coupled with the exaggerated loss of Cpt1a and *Acaa1* expression, may contribute to the increased hepatic steatosis in these animals.

We monitored Cyp isoenzymes that oxidize medium-chain FA. Loss of Nrf2 had no effect on *Cyp2e1* or *Cyp4a10* but significantly reduced *Cyp4a14* expression in livers of RC-fed mice (Fig. 4E). However, the HF diet did not decrease the expression of these Cyp isoenzymes in *Nrf2*^−/−^ livers to levels lower than those observed in *Nrf2*^*+/+*^ livers, so it seems improbable that they account for the higher level of steatosis in mutant livers than in wild-type livers.

### Loss of Nrf2 decreases inhibitory phosphorylation of acetyl-CoA carboxylase.

**FIG 5:**
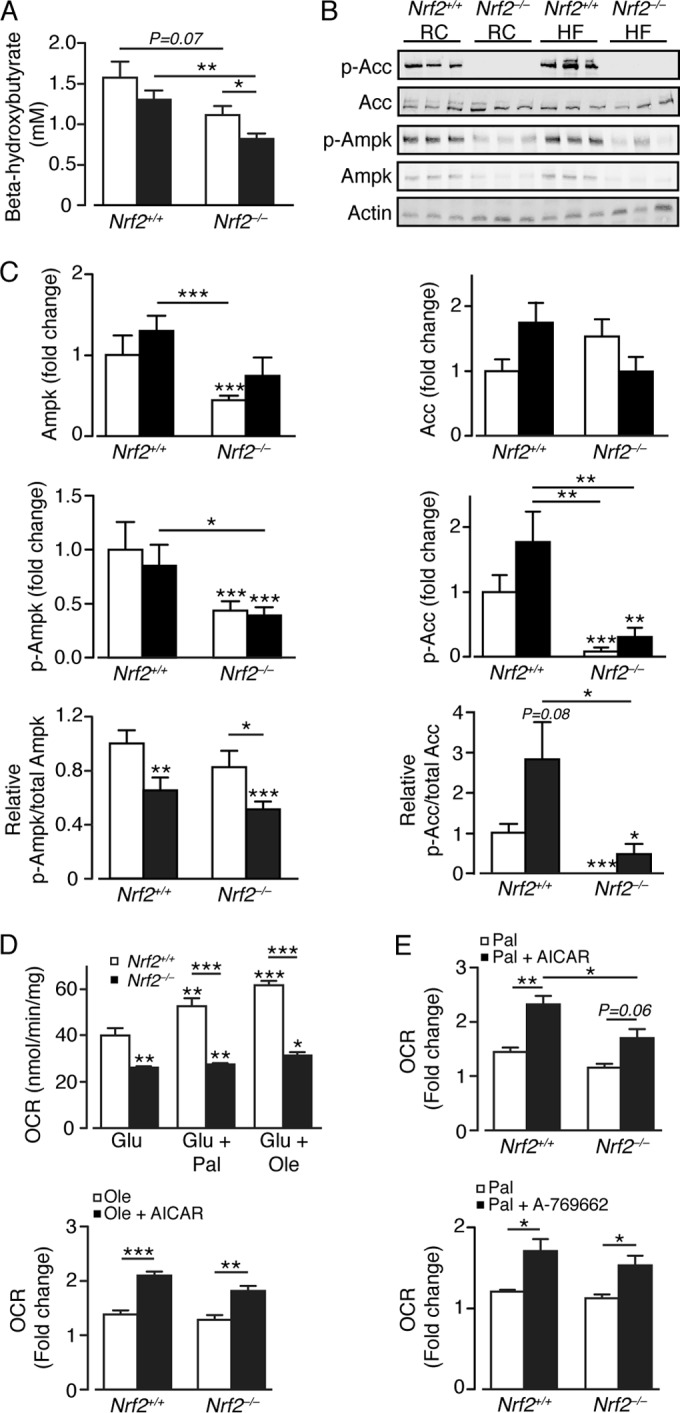
Loss of Nrf2 diminishes phosphorylation of acetyl-CoA carboxylase and decreases the oxidation of free fatty acids. (A) Plasma β-hydroxybutyrate levels in *Nrf2*^*+/+*^ and *Nrf2*^−/−^ mice on an RC or HF diet (9 to 11 mice per group). White bars, RC fed; black bars, HF fed. (B) Representative immunoblots for Acc, p-Acc, Ampk, and p-Ampk, with actin as a loading control, from livers of *Nrf2*^*+/+*^ and *Nrf2*^−/−^ mice on an RC or HF diet. (C) Quantification of the immunoblot data for panel B, with p-Ampk/Ampk and p-Acc/Acc ratios (9 or 10 mice per group). The data were normalized with respect to RC-fed *Nrf2*^*+/+*^ levels for each protein or ratio. White bars, RC fed; black bars, HF fed. (D) OCRs for primary hepatocytes obtained from livers of *Nrf2*^*+/+*^ (*n* = 3) and *Nrf2*^−/−^ (*n* = 4) mice on RC diets, presented with the following substrates: glucose alone (Glu), followed by glucose plus palmitate (Glu + Pal) or glucose + oleate (Glu + Ole). (E) Fold change in OCR on addition of Pal or Ole for primary *Nrf2*^*+/+*^ and *Nrf2*^−/−^ hepatocytes treated or not with AICAR or A-769662. The results are means and SEM. *, *P* < 0.05; **, *P* < 0.01; ***, *P* < 0.001.

The above-mentioned data indicate the HF diet combined with loss of Nrf2 results in decreased hepatic mitochondrial and peroxisomal FA oxidation. One consequence of lowered FA β-oxidation is reduced ketogenesis (82). Consistent with this prediction, RC- and HF-fed *Nrf2*^−/−^ mice exhibited lower plasma β-hydroxybutyrate levels than similarly fed *Nrf2*^*+/+*^ mice (Fig. 5A). FA β-oxidation is regulated by AMPK. Activation of mouse Ampk arises from phosphorylation of Thr172 in its α-subunit, which can lead subsequently to inactivation of AccI and Acc2 by phosphorylation at Ser79 and Ser218, respectively. In turn, inhibition of AccI and Acc2 increases levels of malonyl-CoA, which is a substrate for Fasn and a potent allosteric inhibitor of Cpt1a. Immunoblotting revealed no difference in AccI and/or Acc2 protein (referred to here as Acc) levels by diet or genotype but reduced levels of Ampk protein in *Nrf2*^−/−^ livers (Fig. 5B and C). Moreover, the amounts of phosphorylated Acc (p-Acc) and Ampk (p-Ampk) were reduced in Nrf2-null livers, regardless of diet, with the p-Acc/Acc ratio severely diminished in *Nrf2*^−/−^ compared to *Nrf2*^*+/+*^ livers for both diets. In contrast, the p-Ampk/Ampk ratio was unaltered by genotype and diminished by HF feeding in both *Nrf2*^*+/+*^ and *Nrf2*^−/−^ mouse livers. Thus, loss of Nrf2 diminishes the phosphorylation status of Acc, strongly suggesting elevation in its activity and greater production of malonyl-CoA, an important FA building block and an inhibitor of FA oxidation.

### Nrf2 influences the utilization of glucose and fatty acids in hepatocytes.

We next examined the ability of primary hepatocytes from *Nrf2*^−/−^ and *Nrf2*^*+/+*^ mice to utilize glucose and FA as substrates. Nrf2-null hepatocytes possessed a significantly lower basal glucose oxidation rate and were less able to oxidize palmitate or oleate than *Nrf2*^*+/+*^ hepatocytes (Fig. 5D). Treatment of *Nrf2*^−/−^ hepatocytes with the AMPK activator AICAR or Abbott (A-769662) increased their ability to oxidize palmitate and oleate, proportionally equivalent to that observed for *Nrf2*^*+/+*^ hepatocytes (Fig. 5E). Together, these results indicate that loss of Nrf2 severely diminishes oxidation by the liver of glucose and FA and that this is partly due to a reduction in hepatic Ampk activity in the mouse liver.

### The UPR is perturbed in Nrf2-null mouse liver under basal conditions.

**FIG 6:**
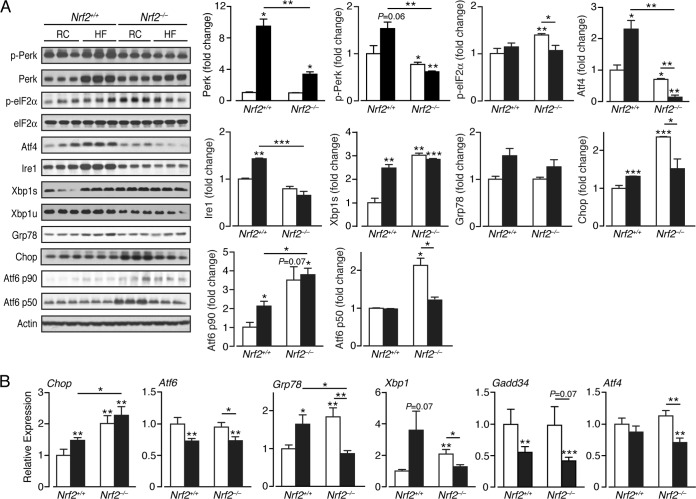
ER stress pathways are perturbed in the livers of *Nrf2*^−/−^ mice. (A) Representative immunoblots from analysis of ER stress and UPR-associated proteins in livers of *Nrf2*^*+/+*^ and *Nrf2*^−/−^ mice on an RC or HF diet. Quantification of the immunoblot data for each protein is shown by genotype and diet (3 mice per group). (B) Quantitative-PCR analysis of mRNAs of ER stress markers from livers of *Nrf2*^*+/+*^ and *Nrf2*^−/−^ mice on RC and HF diets (5 to 8 mice per group). In each case, the data were normalized with respect to RC-fed *Nrf2*^*+/+*^ levels for each protein and gene. White bars, RC fed; black bars, HF fed. The results are means and SEM. *, *P* < 0.05; **, *P* < 0.01; ***, *P* < 0.001.

In *Nrf2*^*+/+*^ livers, the HF diet stimulated a substantial increase in the ER-resident stress sensor proteins Perk and Ire1 (Fig. 6A). Also, an increase in Atf6 p90 but not Atf6 p50 was observed, suggesting the HF diet did not activate the ATF6 pathway in wild-type livers. Stimulation of Perk and Ire1 in *Nrf2*^*+/+*^ livers was associated with induction of Chop and the spliced mRNA form Xbp1s but not Gadd34 (Fig. 6A and B). Remarkably, in RC-fed *Nrf2*^−/−^ livers, substantial increases in Xbp1s and Atf6 p50 were observed, suggesting that the Ire1 and Atf6 ER stress pathways are activated in the mutant mice even under basal conditions. In addition, a modest increase in p-eIF2α, and a more obvious increase in Chop, was observed in RC-fed *Nrf2*^−/−^ livers suggesting that the PERK pathway was also activated, though a decrease in p-Perk and Ire1 was observed relative to the wild-type control. Surprisingly, livers from HF-fed *Nrf2*^−/−^ mice did not exhibit an overt UPR, as evidenced by failure to increase mRNA and/or protein levels of Atf6 p90, Ire1, Atf4, Atf6 p50, Grp78, and Xbp1s compared with HF-fed *Nrf2*^*+/+*^ and/or RC-fed *Nrf2*^−/−^ livers, and we suppose this failure occurs either because the UPR cannot be maintained as a consequence of extensive liver damage or because triggering of the UPR is fundamentally altered in *Nrf2*^−/−^ livers. The only exception to this trend was the observation that Chop mRNA levels were slightly increased in the livers of HF-fed *Nrf2*^−/−^ mice (Fig. 6B).

### Loss of Nrf2 exacerbates inflammation in the liver caused by a high-fat diet.

**FIG 7:**
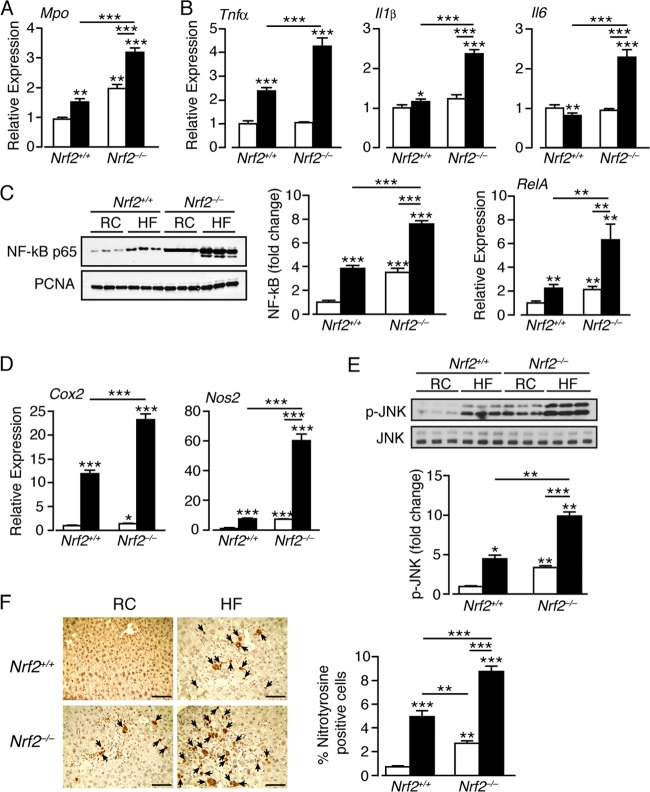
A high-fat diet elicits an exaggerated inflammatory response in the livers of *Nrf2*^−/−^ mice. (A) Quantitative-PCR analysis of mRNA for Mpo in livers of *Nrf2*^*+/+*^ and *Nrf2*^−/−^ mice on an RC or HF diet (10 to 12 mice per group). (B) Quantitative-PCR analysis of levels of mRNAs for the inflammatory markers TNF-α, IL-1β, and IL-6 in livers of *Nrf2*^*+/+*^ and *Nrf2*^−/−^ mice on an RC or HF diet (10 to 12 mice per group). (C) Representative immunoblots for nuclear levels of the p65 subunit of NF-κB in livers of *Nrf2*^*+/+*^ and *Nrf2*^−/−^ mice on an RC or HF diet. PCNA was used as a loading control. Quantification of the immunoblot data for NF-κB p65 is shown (6 mice per group), along with quantitative-PCR analysis of mRNA for RelA (10 to 12 mice per group). (D) Quantitative-PCR analysis of mRNAs for the NF-κB Cox2 and Nos2 target genes in livers of *Nrf2*^*+/+*^ and *Nrf2*^−/−^ mice on an RC or HF diet (10 to 12 mice per group). (E) Representative immunoblot for phosphorylated JNK (p-JNK) and total JNK in livers of *Nrf2*^*+/+*^ and *Nrf2*^−/−^ mice on an RC or HF diet. Quantification of the immunoblot data for p-JNK is shown (3 mice per group). (F) Staining for nitrotyrosine in liver sections from *Nrf2*^*+/+*^ and *Nrf2*^−/−^ mice on an RC or HF diet (the arrows indicate cells giving a positive nitrotyrosine stain; bars, 100 μm). In each case, the data were normalized with respect to RC-fed *Nrf2*^*+/+*^ levels for each protein or gene. White bars, RC fed; black bars, HF fed. The results are means and SEM. *, *P* < 0.05; **, *P* < 0.01; ***, *P* < 0.001.

As activation of PERK and IRE1 during ER stress can stimulate inflammatory responses mediated by NF-κB and JNK (15, 16), we evaluated whether the HF diet differentially stimulates inflammatory responses in wild-type and *Nrf2*^−/−^ livers. First, we measured Mpo, because it is indicative of activated Kupffer cells (65). The HF diet increased Mpo mRNA in both genotypes, with loss of Nrf2 significantly enhancing Mpo expression under both dietary regimes (Fig. 7A). In *Nrf2*^*+/+*^ livers, the HF diet increased mRNAs for the inflammatory markers IL-1β and TNF-α, though it modestly decreased that for IL-6 (Fig. 7B). Compared to wild-type control livers, RC-fed *Nrf2*^−/−^ livers exhibited no increase in TNF-α, IL-1β, and IL-6 expression. However, the HF diet induced their expression to levels significantly higher than it did in *Nrf2*^*+/+*^ mice, suggesting an exacerbated inflammatory response in the knockout mice (Fig. 7B).

The nuclear abundance of the NF-κB p65 RelA subunit was increased in livers of HF-fed *Nrf2*^*+/+*^ and RC-fed *Nrf2*^−/−^ mice, with the HF diet raising NF-κB p65 levels in *Nrf2*^−/−^ livers even further (Fig. 7C). The HF diet also produced pronounced increases in RelA mRNA in *Nrf2*^−/−^ mice (Fig. 7C). The expression of the NF-κB target genes Cox2 and Nos2 was increased by 12- and 6-fold, respectively, in HF-fed *Nrf2*^*+/+*^ livers and by 23- and 60-fold, respectively, in livers of HF-fed *Nrf2*^−/−^ mice (Fig. 7D). We also measured JNK activation. As expected, p-JNK levels were increased by the HF diet in *Nrf2*^*+/+*^ livers and were elevated in RC-fed *Nrf2*^−/−^ livers. Significantly, p-JNK levels were highest in *Nrf2*^−/−^ livers (Fig. 7E).

As Nos2 was highly expressed in HF *Nrf2*^−/−^ livers, it is possible that excessive nitric oxide levels contribute to inflammation. We therefore examined the possibility that nitrotyrosine levels were high in livers from *Nrf2*^−/−^ mice. As anticipated, staining for nitrotyrosine was greater in HF *Nrf2*^−/−^ livers than in the other livers examined (Fig. 7F).

Collectively, these results indicate that the HF diet stimulates a mild inflammatory response in livers of *Nrf2*^*+/+*^ mice. However, *Nrf2*^−/−^ mice exhibited mild liver inflammation even under basal conditions, and this was markedly aggravated by the HF diet.

### Livers from high-fat-fed *Nrf2*^−/−^ mice are subject to oxidative stress and hepatocellular injury.

**FIG 8:**
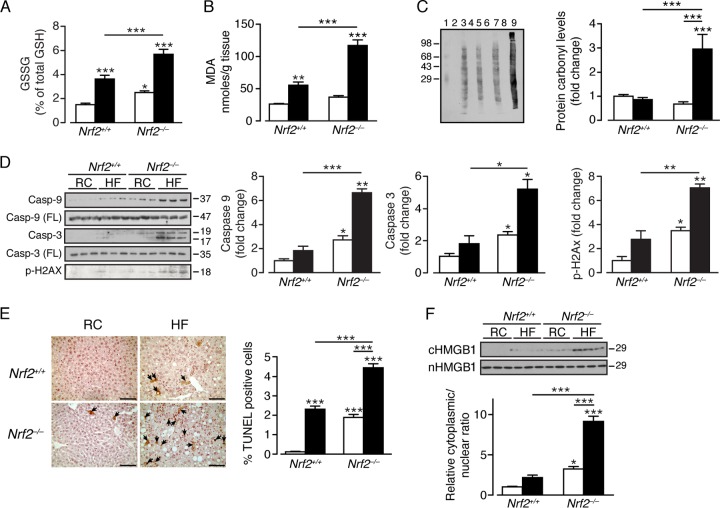
The livers of *Nrf2*^−/−^ mice fed a high-fat diet are subject to oxidative stress and exhibit an increased level of apoptosis and DNA damage. (A) Ratio of GSSG to GSH in the livers of *Nrf2*^*+/+*^ and *Nrf2*^−/−^ mice on an RC or HF diet (6 mice per group). (B) Lipid peroxidation product MDA levels in livers of *Nrf2*^*+/+*^ and *Nrf2*^−/−^ mice on an RC or HF diet (8 mice per group). (C) Levels of oxidized protein shown as a representative OxyBlot using 20 μg of protein from each experimental group. Lanes: 1, molecular weight standard; 2, 4, 6, and 8, negative controls (no 2,4-dinitrophenylhydrazine); 3, RC-fed *Nrf2*^*+/+*^; 5, RC-fed *Nrf2*^−/−^; 7, HF-fed *Nrf2*^*+/+*^; 9, HF-fed *Nrf2*^−/−^. The graph shows densitometric measurements of lanes 3, 5, 7, and 9 (normalized to the RC-fed *Nrf2*^*+/+*^ amount) in livers of *Nrf2*^*+/+*^ and *Nrf2*^−/−^ mice on an RC or HF diet (8 to 11 mice per group). (D) Representative immunoblots of full-length (FL) and cleaved caspase 9 and caspase 3 and phosphorylated H2AX in livers of *Nrf2*^*+/+*^ and *Nrf2*^−/−^ mice on an RC or HF diet. (E) DNA fragmentation in livers of *Nrf2*^*+/+*^ and *Nrf2*^−/−^ mice on an RC or HF diet was examined by TUNEL assay (the arrows indicate apoptotic/necrotic cells; bars, 100 μm), with the mean percentages of TUNEL-positive cells shown (5 mice per group). (F) Immunoblot for HMGB1 in nuclear and cytosolic fractions of *Nrf2*^*+/+*^ and *Nrf2*^−/−^ livers from RC- or HF-fed mice. Quantification of the immunoblot data for each protein is shown by genotype and diet (3 mice per group). In each case, the data were normalized with respect to RC-fed *Nrf2*^*+/+*^ levels for each protein. White bars, RC fed; black bars, HF fed. The results are means and SEM. *, *P* < 0.05; **, *P* < 0.01; ***, *P* < 0.001. Molecular mass markers (C, D, and F) are in kDa.

We examined whether the HF diet provokes oxidative stress in the liver by measuring GSH, GSSG, MDA, and the levels of oxidized protein. In *Nrf2*^*+/+*^ mice, the HF diet produced an increase in GSSG (Fig. 8A) and MDA (Fig. 8B), but no change was observed in protein carbonyl levels, suggesting that the extent of oxidative stress in wild-type mice was not sufficient to cause irreversible damage (Fig. 8C). In comparison, the livers of RC-fed *Nrf2*^−/−^ mice contained significantly increased GSSG (Fig. 8A), but the levels of MDA and protein carbonyls did not differ from those in RC-fed *Nrf2*^*+/+*^ livers. Most strikingly, the HF-fed *Nrf2*^−/−^ livers contained elevated GSSG and large increases in MDA and protein carbonyls compared with either RC- or HF-fed *Nrf2*^*+/+*^ mice (Fig. 8A to C), indicating that the higher oxidative-stress load produced by the HF diet in the mutant mouse exceeded its intrinsic antioxidant capacity.

The *Nrf2*^−/−^ livers exhibited evidence of apoptosis and necrosis. Thus, in HF-fed *Nrf2*^−/−^ livers but not HF-fed *Nrf2*^*+/+*^ livers, increases in cleaved caspases 3 and 9 were observed, as was an increase in a marker for DNA double-strand breaks, p-H2AX (Fig. 8D). RC-fed *Nrf2*^−/−^ mouse livers also demonstrated evidence of apoptosis, with increased levels of cleaved caspases 3 and 9 and p-H2AX. TUNEL analysis revealed a marked increase in DNA fragmentation in HF-fed *Nrf2*^*+/+*^ and RC-fed *Nrf2*^−/−^ livers and that cleaved DNA was present in the cytoplasm of a substantial number of hepatocytes (Fig. 8E). This pattern of staining is consistent with necrotic cell death and was observed to a much greater degree in HF-fed *Nrf2*^−/−^ livers. Immunoblotting for the nuclear nonhistone protein HMGB1 in cytosolic fractions also suggested release in RC- and HF-fed *Nrf2*^−/−^ livers of nuclear protein into the cytoplasm, which suggests loss of nuclear membrane integrity in a significant portion of hepatocytes that lack Nrf2 (Fig. 8F).

### Nrf2^−/−^ livers fail to adapt to high-fat diet-stimulated oxidative stress.

**FIG 9:**
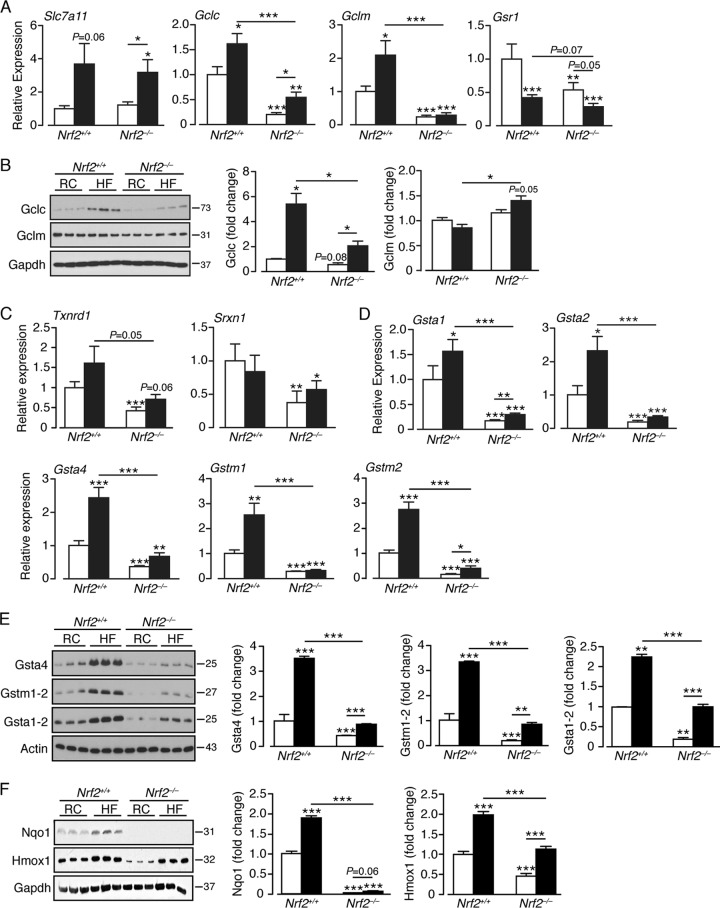
A high-fat diet stimulates an antioxidant response in *Nrf2*^*+/+*^ livers that is attenuated in *Nrf2*^−/−^ livers. (A) Quantitative-PCR analysis of mRNAs for glutathione homeostasis genes in livers of *Nrf2*^*+/+*^ and *Nrf2*^−/−^ mice on an RC or HF diet (7 to 12 mice per group). (B) Representative immunoblots of Gclc and Gclm proteins in livers of *Nrf2*^*+/+*^ and *Nrf2*^−/−^ mice on an RC or HF diet. Quantification of the immunoblot data for each protein is shown by genotype and diet (3 mice per group). (C) Quantitative PCR of mRNAs for Txnrd1 and Srxn1 in livers of *Nrf2*^*+/+*^ and *Nrf2*^−/−^ mice on an RC or HF diet. (D) Quantitative PCR of mRNA for “prototypic” ARE-driven genes from livers of *Nrf2*^*+/+*^ and *Nrf2*^−/−^ mice on an RC or HF diet (10 to 12 mice per group). (E) Representative immunoblots of Gsta4, Gstm1-2, and Gsta1-2 in livers of *Nrf2*^*+/+*^ and *Nrf2*^−/−^ mice on an RC or HF diet. Quantification of the immunoblot data for each protein is shown by genotype and diet (3 mice per group). (F) Representative immunoblots of Nqo1 and Hmox1 in livers of *Nrf2*^*+/+*^ and *Nrf2*^−/−^ mice on an RC or HF diet. Quantification of the immunoblot data for each protein is shown by genotype and diet (6 mice per group). In each case, the data were normalized with respect to RC-fed *Nrf2*^*+/+*^ levels for each protein or gene. White bars, RC fed; black bars, HF fed. The results are means and SEM. *, *P* < 0.05; **, *P* < 0.01; ***, *P* < 0.001. Molecular mass markers (B, E, and F) are in kDa.

Consistent with the notion that the HF diet elicits an antioxidant response (37, 39), mRNA levels for the cystine/glutamate antiporter Slc7a11 and the Gclc and Gclm subunits, which together catalyze the rate-limiting step in glutathione synthesis, were increased in HF-fed *Nrf2*^*+/+*^ livers (Fig. 9A). An increase in mRNA for Gsr1, which catalyzes reduction of GSSG to GSH, was not observed. The HF diet also increased the amount of Gclc protein but not Gclm protein in *Nrf2*^*+/+*^ livers (Fig. 9B). In contrast, RC-fed *Nrf2*^−/−^ livers contained smaller amounts of mRNA for Gclc and Gclm than their *Nrf2*^*+/+*^ counterparts, and also reduced levels of Gclc protein, with diminished or no increase following administration of the HF diet, suggesting that the basal expression of these genes is compromised in the mutant mice and that they mount a blunted antioxidant response. An exception to this outcome was the level of Gclm protein, which was relatively unchanged by genotype and exhibited a small increase in response to the HF diet. The failure of Gclm protein to reflect changes in Gclm mRNA is probably due to posttranscriptional regulation of the subunit, but at present, no definitive explanation exists for this anomaly. The hypothesis that *Nrf2*^−/−^ livers have less intrinsic antioxidant capacity than *Nrf2*^*+/+*^ livers is supported by the fact that levels of mRNAs for Txnrd1, which reduces oxidized thioredoxin, and Srxn1, which reactivates overoxidized peroxiredoxin, are significantly lower in RC- and HF-fed *Nrf2*^−/−^ livers (Fig. 9C).

To examine whether the HF diet induces prototypic ARE-driven genes, we measured Gst subunit mRNA levels and Gst, Nqo1, and Hmox1 proteins. In *Nrf2*^*+/+*^ livers, the HF diet produced ∼2- to 3-fold increases in mRNA levels for Gsta1, Gsta2, Gsta4, Gstm1, and Gstm2 and similar increases in the amounts of Gsta1/2, Gsta4, Gstm1/2, Hmox1, and Nqo1 proteins (Fig. 9C to E), suggesting that livers of wild-type mice can adapt to the oxidative stress presented by consumption of an HF diet. As expected, in RC-fed *Nrf2*^−/−^ mouse livers, levels of these mRNAs and proteins were lower than in RC-fed *Nrf2*^*+/+*^ livers, indicating that Nrf2 controls their basal expression. However, the HF diet stimulated a modest induction of the majority of these genes in the mutant mice, albeit from a lower basal level (Fig. 9D to F), suggesting that *Nrf2*^−/−^ livers are intrinsically more sensitive to oxidative stress and are less able to adapt to such insult.

## DISCUSSION

NASH may arise through a “two-step” process in which insulin resistance first produces steatosis and subsequently oxidative stress drives inflammation (66, 67). As loss of Nrf2 increases both expression of lipogenesis genes and sensitivity to oxidative stress, it seems likely to contribute to the etiology of NASH. We therefore examined whether *Nrf2*^−/−^ mice become insulin resistant when fed an HF diet and whether this results in their being more sensitive than wild-type mice to developing NASH.

### The development of NASH in *Nrf2*^−/−^ mice does not require insulin resistance.

Upon HF feeding, we found *Nrf2*^−/−^ mice exhibit partial protection against obesity, increased energy expenditure, and better glucose disposal and insulin sensitivity than HF-fed *Nrf2*^*+/+*^ mice. Such outcomes, although conflicting with the majority of Nrf2 gain-of-function studies (37, 68, 69), are in agreement with other recent studies of long-term HF-fed *Nrf2*^−/−^ mice (39, 43). The increased strength of insulin signaling in both *Nrf2*^−/−^ livers and skeletal muscle possibly reflects impaired antioxidant capacity in the mutant animal that results in an increase in H_2_O_2_ levels, which in turn increases inactivation of protein tyrosine phosphatase 1B that antagonizes insulin signaling (70). The situation in *Nrf2*^−/−^ mice may resemble that in glutathione peroxidase 1 knockout mice, where a relative decrease in the ability to eliminate H_2_O_2_ enhances insulin sensitivity (71). Although further work is required to demonstrate why HF-fed *Nrf2*^−/−^ mice are more sensitive to insulin than their wild-type counterparts, our study demonstrates clearly that insulin resistance is not a prerequisite for NASH in *Nrf2*^−/−^ mice. This is an unexpected result, because it is commonly believed that ER stress and inflammation, both of which occur in *Nrf2*^−/−^ livers, are inexorably linked to insulin resistance (16).

### Steatosis in *Nrf2*^−/−^ mice is associated with multiple changes in lipid metabolism genes.

The greater degree of steatosis in livers of *Nrf2*^−/−^ mice fed the HF diet for 24 weeks than in livers of similarly treated *Nrf2*^*+/+*^ mice is multifactorial. In part, it probably arises because the mutant animals upregulate the FA uptake protein Fabp5 and the FA synthesis enzymes Acly, Acaca, Fasn, and Elovl6 to a greater extent than *Nrf2*^*+/+*^ mice. It is also likely that the greater decrease in Cpt1a, which is rate limiting for FA β-oxidation, in *Nrf2*^−/−^ livers than in *Nrf2*^*+/+*^ livers contributes to increased steatosis in the mutant animals.

The heightened upregulation of Fabp5, Acly, Acaca, Fasn, and Elovl6 in *Nrf2*^−/−^ livers in response to the HF diet is likely to be due to the increased expression of the transcription factors Srebf1, Srebf2, Mlxipl, and PPARγ compared with that in HF *Nrf2*^*+/+*^ livers. Furthermore, decreased expression of Shp, which inhibits the cholesterol and FA homeostasis transcription factor LXRα, may also contribute to steatosis. Consistent with the latter idea, Kay et al. (45) reported that loss of Nrf2 greatly exacerbates stimulation of NASH by the LXRα agonist T0901317, whereas activation of Nrf2 by administration of sulforaphane inhibited NASH caused by the LXRα agonist. Indeed, they proposed that activation of Nrf2 resulted in FXR-mediated induction of Shp, which subsequently inhibited LXRα by forming an inactive LXRα-Shp heterodimer, and prevented T0901317 from stimulating NASH.

### AMPK signaling is impaired in *Nrf2*^−/−^ mice.

An unexpected finding was that Acc is no longer subject to inhibitory phosphorylation in the livers of RC- and HF-fed *Nrf2*^−/−^ mice. The resulting increase in Acc is likely to augment malonyl-CoA levels, thus driving lipogenesis and decreasing FA oxidation. This was a surprising result, because AMPK, the kinase predominantly responsible for inhibitory Acc phosphorylation, is activated by H_2_O_2_ through phosphorylation by LKB1 (72, 73), and it is probable that H_2_O_2_ is more abundant in *Nrf2*^−/−^ than in *Nrf2*^*+/+*^ livers. Immunoblotting suggested that the lack of Acc phosphorylation in *Nrf2*^−/−^ livers results from reduction in AMPK activity. However, treatment with the AMPK activators AICAR and A-769662 enhanced the ability of *Nrf2*^−/−^ hepatocytes to oxidize FA, suggesting the kinase can be activated in livers from knockout mice. Further work is required to establish how Nrf2 regulates this kinase. Intriguingly, the findings that *Ampkβ1*^−/−^ mice are protected from HF diet-induced steatosis (74) and that Ampkβ1 (i.e., Prkab1) is upregulated in *Nrf2*^−/−^ mice (31) suggest that it may contribute to exacerbation of steatosis in *Nrf2*^−/−^ liver. Furthermore, reduced Ampk activity in *Nrf2*^−/−^ livers also results in increased activity of Srebp-1c and Srebp-2, which drive lipogenesis.

### Nrf2 suppresses ER stress and inflammation.

We found that Xbp1s, Chop, p-elf2α, and Atf6 protein levels and Chop, Xbp1, and Grp78 transcripts are upregulated in the livers of RC-fed *Nrf2*^−/−^ mice, suggesting they may experience ER stress under basal conditions. It has not been reported previously that *Nrf2*^−/−^ livers are subject to ER stress, but as we examined mice that are 32 to 34 weeks of age, it is possible this phenotype is apparent only in older mice, not the younger animals that are more commonly studied. Notably, *Nrf2*^−/−^ fibroblasts have previously been reported to upregulate Chop under basal conditions (75), which agrees with our findings.

As ROS facilitates triggering of the UPR, and the antioxidant butylated hydroxyanisole can reduce ER stress (76, 77), it might be anticipated that *Nrf2*^−/−^ mice are susceptible to ER stress. Evidence suggests that the UPR, along with ROS and release of Ca^2+^ from the ER, is linked to inflammation through activation of NF-κB and JNK (15, 16). Therefore, the increase in NF-κB and p-JNK proteins and expression of Cox2 and Nos2 in RC-fed *Nrf2*^−/−^ livers is likely partly due to constitutive activation of the UPR in livers of mutant mice. Furthermore, the overexpression of Chop in *Nrf2*^−/−^ livers may contribute to metabolic dysregulation, inflammation, and fibrosis, as these conditions have been linked to increased Chop levels in the mouse (78).

Our finding that the nuclear protein HMGB1 is present in the cytosolic fraction of HF *Nrf2*^−/−^ livers is interesting, because upon release from cells, it can function as a damage-associated molecular-pattern polypeptide that contributes to inflammatory processes (79, 80). Importantly, signaling by HMGB1 is influenced by its redox status, and this might be expected to differ markedly in *Nrf2*^−/−^ and wild-type mice.

Surprisingly, in HF-fed *Nrf2*^−/−^ livers there was an overall reduction in the levels of ER stress markers. This apparent failure to maintain the UPR in livers of HF-fed *Nrf2*^−/−^ mice might be associated with chronic damage owing to the duration of the experiment. Further experiments are required to allow the time course of HF diet-stimulated ER stress in wild-type and *Nrf2*^−/−^ livers to be documented.

### Concluding comments.

The results presented here show that knockout of Nrf2 renders mice more sensitive to NASH when placed on an HF diet. Our data indicate that the increase in steatosis in *Nrf2*^−/−^ mice placed on the HF diet is due to a heightened level of induction of lipogenesis genes and suppression of β-oxidation genes compared with wild-type animals. Administration of the HF diet stimulated hepatic oxidative stress, and as livers of *Nrf2*^−/−^ mice are unable to adapt to such insults, this leads to failure to maintain normal homeostatic levels of the glutathione- and thioredoxin-based antioxidant systems, which may stimulate inflammation. Unexpectedly, we found that the UPR is perturbed in the livers of *Nrf2*^−/−^ mice on the RC control diet, suggesting that ER stress is triggered in these animals even under basal conditions. It is therefore possible that disturbance of the UPR in the livers of *Nrf2*^−/−^ mice contributes to the rapid development of NASH when they are placed on an HF diet. In humans, promoter polymorphisms exist in the *NRF2* gene (*NFE2L2*) that alter its activity (81), and it will be interesting in the future to determine whether they influence lipid metabolism, oxidative stress, inflammation, or ER stress in humans.
